# The effect of modulators on lung function following inpatient treatment for CF exacerbations

**DOI:** 10.3389/fped.2025.1654122

**Published:** 2025-08-21

**Authors:** Anne Stone, Meredith B. Haag, Corinne A. Muirhead, Sheila Markwardt, Kelvin D. MacDonald

**Affiliations:** ^1^ Division of Pediatric Pulmonology, Department of Pediatrics, Oregon Health & Science University, Portland, OR, United States; ^2^ Division of Neonatology, Department of Pediatrics, Oregon Health & Science University, Portland, OR, United States; ^3^ Department of Pharmacy Services, Oregon Health & Science University, Portland, OR, United States; ^4^ Biostatistics and Design Program, Oregon Health and Science University, Portland, OR, United States

**Keywords:** lung function, cystic fibrosis, CFTR modulator drugs, adolescent and young adult (AYA), pulmonary exacerbation

## Abstract

**Background:**

Modulator therapy restores CFTR function and has led to health benefits for persons with cystic fibrosis (CF) (PwCF), including lower rates of pulmonary exacerbations. It is unknown if modulators affect lung function trajectories after inpatient treatment of pulmonary exacerbations (PEx).

**Methods:**

We conducted a retrospective review of hospital encounters for PEx for subjects 6–25 years old with mild to moderate lung disease admitted to a large tertiary care center from 2014 to 2021 to capture hospitalizations of PwCF before and after starting modulators. Descriptive analyses were used to characterize the population and lung function findings. Logistic regression analyses were conducted to assess the association between modulators and FEV1pp outcomes.

**Results:**

The study sample included 575 encounters representing 149 unique PwCF. Hospital encounters of PwCF taking modulator were associated with higher mean FEV1pp at baseline, midway, discharge, and follow-up assessments. Mean FEV1pp increased during inpatient treatment of PEx with loss of lung function at follow-up, regardless of modulator use. Hospital encounters of PwCF not taking modulators were associated with less significant improvements in mean FEV1pp from admission at both midway and discharge (15.6% vs. 18.3%, 19.9% vs. 22.5%, no modulator vs. modulator groups, respectively). At follow-up, hospitalizations of PwCF taking modulators were associated with a significantly higher probability of sustained improvement in FEV1pp from discharge (difference in probabilities 0.119, *p* < 0.05) and a lower probability of loss of lung function from baseline (difference in probabilities −0.123, *p* < 0.05).

**Conclusions:**

Hospitalizations for PwCF taking modulators were associated with higher lung function at all assessments. Hospitalizations of PwCF taking modulators were associated with a significantly higher probability of sustained improvement in FEV1pp following discharge and a lower probability of loss of lung function from baseline.

## Introduction

Cystic fibrosis transmembrane regulator (CFTR) modulator therapy restores CFTR function and has led to multiple health benefits for persons with CF (PwCF) (1–9). Modulators have improved lung function and nutritional status and decreased respiratory symptoms (2–9). Moreover, the rates of pulmonary exacerbations are significantly lower among PwCF on modulator therapy (2–5, 8, 9). It is unknown if modulators also affect lung function trajectories during and after inpatient treatment of pulmonary exacerbations (PEx).

All modulators are associated with significantly lower rates of PEx among PwCF. Recurrent PEx remain a driver of lung function decline and morbidity (10–13). Duration of treatment for PEx is variable, with a median duration of approximately 14 days based on current understandings of expected lung function recovery (14–16). Limited data suggest that higher baseline lung function is associated with a shorter time to lung function recovery during inpatient therapy among PwCF with chronic *Pseudomonas aeruginosa* (PA) infection (15). Prior work revealed that two-drug combination modulator therapy (lumacaftor/ivacaftor and tezacaftor/ivacaftor) in adults did not lead to greater lung function recovery during PEx compared with modulator-naïve controls (17). This study aimed to determine the effects of any CFTR modulator on the ability to recover and sustain improvements in lung function in children and young adults with CF after inpatient treatment for PEx. We hypothesized that any modulator improves lung function trajectories during and after treatment of PEx.

## Materials and methods

### Study sample

We conducted a retrospective chart review of hospital encounters for PEx for PwCF admitted to a tertiary teaching hospital with a 120,000 square mile catchment area including Oregon and Southern Washington from 2014 to 2021 to capture PEx treatment encounters for PwCF before and after taking modulators. A waiver of informed consent was granted by the institutional review board (IRB) at Oregon Health & Science University (IRB 00023009) since this study only examined historical data and the number of charts reviewed rendered the contact of each individual impracticable. Encounters for subjects with baseline forced expiratory volume in 1 s (FEV1) <50% predicted, CF-related diabetes (CFRD), allergic bronchopulmonary aspergillosis, history of lung transplant, non-invasive positive pressure ventilation, or active non-tuberculosis mycobacteria infection were excluded due to their potential association with a negative influence on FEV1pp outcomes during and after PEx and their potential association with more modest clinical benefits. Other factors, such as positive sputum culture for methicillin-resistant *Staphylococcus aureus* or PA, were included since they are common among hospitalized PwCF taking and not taking modulators and therefore generalizable to a broad CF population. Hospital encounters for PEx shorter than 8 days and/or without any assessments of lung function were excluded. Hospitalizations that began at outside hospitals and led to transfer or included treatment courses completed at home were included.

### FEV1 percent predicted (FEV1pp)

Lung function outcomes were reported as percent predicted values using NHANES race-based normative data and measured at the following timepoints: baseline, admission, 5 or more days post-admission (midway), discharge, and follow-up. Baseline lung function was defined as the best forced expiratory volume in 1 s percent predicted (FEV1pp) in the year prior to the hospital encounter. Admission lung function included assessments from a clinic visit in which PEx was diagnosed or after hospital admission if completed within the first 3 days of hospitalization. Midway lung function was defined as the first assessment completed 5 or more days after hospitalization. Discharge lung function was defined as assessments within 3 days of hospital discharge. If the midway assessment was completed within 3 days of hospital discharge, values from this assessment were reported as both the midway and discharge values. Follow-up lung function was defined as assessments completed one month or more following hospitalization. All FEV1 assessments were completed prior to bronchodilator administration.

### FEV1pp outcome measures

Significant change in lung function was defined as a relative ≥10% change in FEV1pp. A significant decline at admission was a ≥10% drop from baseline, significant improvement at midway was a ≥10% improvement from admission, significant improvement at discharge was a ≥10% improvement from admission, sustained improvement was ≤10% decline from discharge at follow-up, and significant loss of lung function was a ≥10% decline from baseline at follow-up.

### Modulators

In this study, we compare lung function assessments during hospital encounters of PwCF taking any modulator therapy to lung function assessments during hospital encounters of PwCF not taking modulator therapy. If a modulator was started at any point during a hospital encounter, the encounter was included as a hospitalization of a PwCF on modulator therapy.

### Demographic and additional clinical characteristics

Demographic and clinical data were abstracted from the electronic health record (EHR). These include biologic sex (male vs. female), race/ethnicity, genotype (dF508/dF508, dF508/other, vs. other/other), age at admission, body mass index (BMI; calculated as weight/height^2^), and consecutive sputum culture results over the year prior. Additionally, data about the hospital encounter were collected, including admission and discharge dates, length of stay, use of systemic steroids, and FEV1pp outcomes at different timepoints.

### Statistical analysis

Descriptive statistics were used to summarize characteristics at the time of a PEx according to modulator use (yes vs. no); frequencies and percentages were calculated for categorical variables, while means and standard deviations were calculated for continuous variables.

Initial assessment of FEV1pp in our study sample included a descriptive summary of FEV1pp at each time point and percent change in FEV1pp between key timepoints. The prevalence of each FEV1pp outcome was calculated for the overall study cohort and by modulator use as well as stratified by age (5–12 years and 12+ years).

We used logistic regression to assess the association between modulator therapy and FEV1pp outcomes, which were all binary (yes vs. no). Since modulator therapy was not randomly assigned at each patient encounter, we weighted our regression models with an inverse probability of treatment weight (IPTW), which accounts for potential confounding, specifically confounding by indication, and selection bias. The following variables were selected *a priori* to be included in our propensity score models: age at admission, sex, race/ethnicity (white vs. non-white), baseline FEV1pp, percent change from baseline at admission, BMI, previous PEx admissions, PA-positive sputum culture, and steroid treatment (yes vs. no). These include variables unrelated to modulator therapy use but potentially related to exacerbation severity and/or FEV1pp outcomes, since inclusion of such variables may increase the precision of the estimated treatment effect without increasing bias (18). As a PwCF may have been represented more than once in our study sample, leading to a potential correlation of encounters within a patient, a cluster robust variance estimator was used in all IPTW regression models to account for the possible repeated encounters within an individual PwCF.

Three sensitivity analyses were performed to examine the robustness of our results. These included building IPTW logistic regression models as described above for (1) patients with dF508 homozygous genetics only because they represent a CF population with the most potential for CFTR rescue with modulator therapy (19), (2) encounters with a midway assessment performed at least 3 days prior to discharge, and (3) encounters representing all but 13 high-utilizing, potentially influential patients. All analyses were conducted in Stata/SE, version 15 (StataCorp, College Station, TX, United States).

## Results

**Figure 1:**
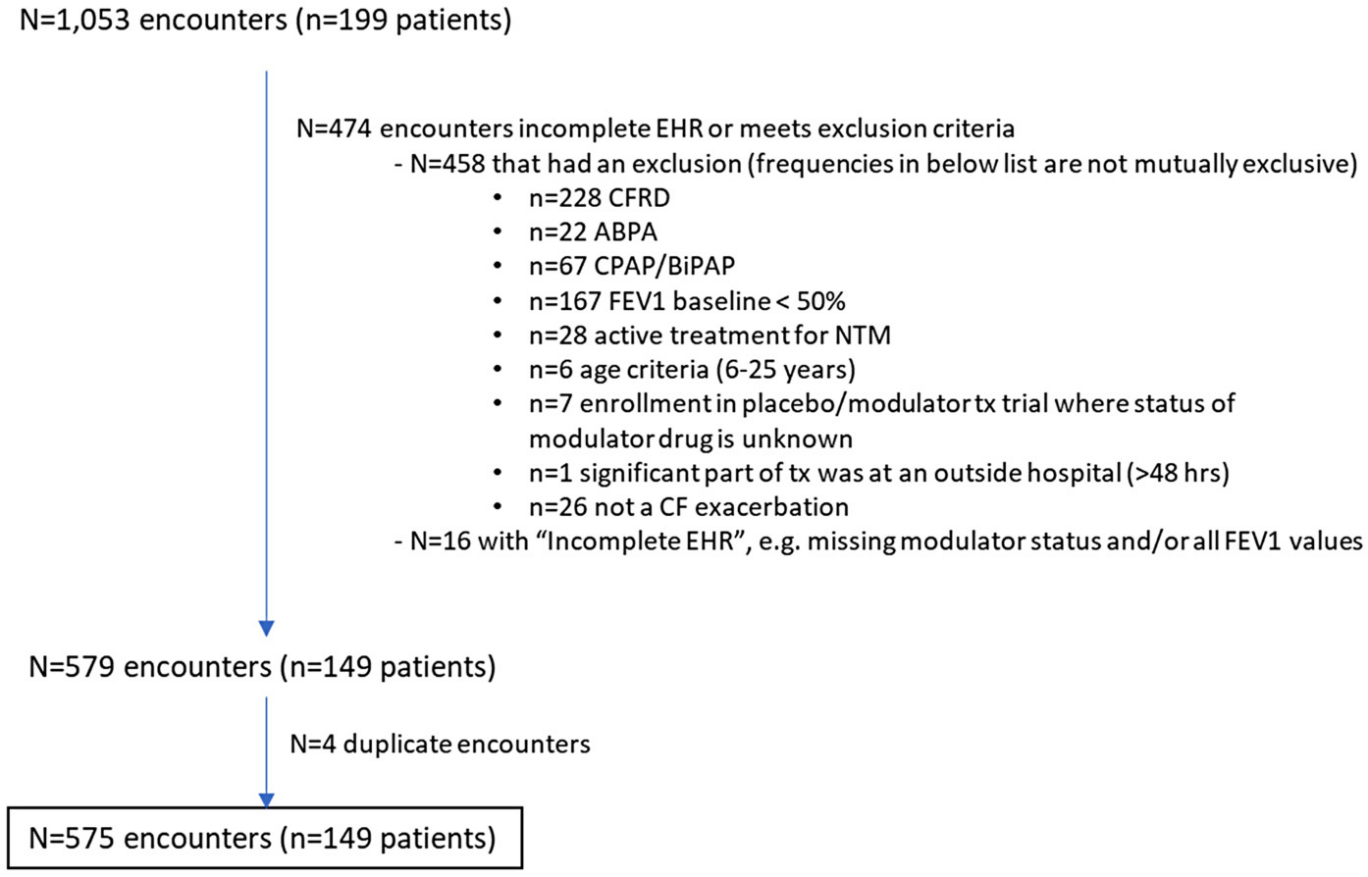
Study cohort diagram.

Data from a total of 5,798 hospital encounters for 362 unique subjects were extracted from the EHR. Data review identified 1,053 encounters of 199 subjects. Of these, 458 met exclusion criteria (most common exclusion criteria were CFRD and FEV1pp <50%), and 16 had incomplete data, leaving 579 encounters. An additional four encounters were identified as duplicates, leaving 575 encounters for PEx for 149 PwCF aged 6–25 years (see Figure 1 for CONSORT-like diagram). Of the 149 PwCF, 76 (51%) were male, 90 (60%) were dF508 homozygous, and 44% used a modulator at some point during the study period. Availability of various modulators in the United States during the study period can be seen in Supplementary Table S1. Characteristics of the 575 hospital encounters by modulator type are seen in Table 1. In all, 152 hospital encounters were of PwCF taking modulator therapy. Of these, only 26 (17%) were of PwCF taking either ivacaftor or elexacaftor/tezacaftor/ivacaftor plus ivacaftor (ETI), modulators associated with higher CFTR restoration. There was wide variability in the length of time since admission for which a modulator had been taken (0–4,697 days), with some PwCF starting modulator therapy during a hospital encounter. Hospital encounters for PwCF taking modulator vs. not taking modulator differed significantly by sex and genotype, but were similar in rates of colonization of PA, use of systemic steroids, the number of hospitalizations for PEx in the previous year, and length of stay. Body mass index was significantly higher in hospitalizations of PwCF taking modulators.

**Table 1:** Characteristics at time of exacerbation by modulator use (yes vs. no) (*n* = 575 hospitalizations).

Characteristic	Modulator therapy		*p*-value^a^
	No (*n* = 423)	Yes (*n* = 152)	
Modulator therapy characteristics			
Modulator type, *n* (%)			
None	423 (100.0%)	0 (0.0%)	
Ivacaftor	—	6 (3.9%)	
Lumacaftor/ivacaftor	—	97 (63.8%)	
Tezacaftor/ivacaftor + ivacaftor	—	29 (19.1%)	
Elexacaftor/tezacaftor/ivacaftor + ivacaftor	—	20 (13.2%)	
Time on modulator (days), median (IQR) [range]	—	416.5 (135.0–754.0) [0–4,697]	
Time on modulator, categorical, *n* (%)			
≤30 days	—	16 (10.5%)	
1–6 months	—	28 (18.4%)	
6 months to 1 year	—	22 (14.5%)	
1–2 years	—	47 (30.9%)	
2+ years	—	39 (25.7%)	
Patient characteristics			
Male sex, *n* (%)	178 (42.1%)	89 (58.6%)	<0.001
Race/Ethnicity, *n* (%)			0.570
American Indian	2 (0.5%)	2 (1.3%)	
Asian	1 (0.2%)	0 (0.0%)	
Black	3 (0.7%)	0 (0.0%)	
Hispanic	34 (8.0%)	10 (6.6%)	
White	383 (90.5%)	140 (92.1%)	
Sputum culture, *n* (%)			
Methicillin-sensitive *S. aureus*	258 (61.0%)	82 (53.9%)	0.130
Methicillin-resistant *S. aureus*	112 (26.5%)	41 (27.0%)	0.910
*Haemophilus influenzae*	119 (28.1%)	46 (30.3%)	0.620
*Pseudomonas aeruginosa*	241 (57.0%)	92 (60.5%)	0.450
*Stenotrophomonas maltophilia*	81 (19.1%)	36 (23.7%)	0.230
*Achromobacter* species	61 (14.4%)	26 (17.1%)	0.430
*Burkholderia* species	18 (4.3%)	10 (6.6%)	0.250
*Aspergillus* species	129 (30.5%)	39 (25.7%)	0.260
Non-tuberculous mycobacteria	14 (3.3%)	6 (3.9%)	0.710
Hospitalization characteristics			
Steroid treatment, *n* (%)	105 (24.8%)	30 (19.7%)	0.200
Previous PEx admissions, median (IQR) [range]	3.0 (2.0–6.0) [1–7]	3.0 (2.0–7.0) [1–7]	0.630
Length of stay (days), median (IQR) [range]	13.0 (10.0–15.0) [6–27]	13.0 (10.0–15.0) [0–24]	0.550
Home therapy, *n* (%)	77 (18.2%)	25 (16.6%)	0.650
Time to follow-up, days, median (IQR) [range]	41 (28–70.5) [7–396]	42 (29–78) [7–361]	0.726

^a^ *p*-values are from chi-square or Fisher's exact tests for categorical variables and two-sample *t*-tests for continuous variables.

Mean lung function at different time points related to the hospital encounter by use of modulator therapy is shown in Table 2. Hospital encounters of PwCF taking modulator appeared to have higher mean FEV1pp at baseline, midway, discharge, and follow-up, but this was not statistically analyzed. Mean FEV1pp at admission was similar regardless of modulator use. Mean FEV1pp increased during inpatient treatment of PEx for encounters regardless of modulator use.

**Table 2:** Mean and standard deviation of FEV1pp at key timepoints, overall and by modulator use (yes vs. no).

Timepoint	Total (*N* = 575)	Modulator therapy	
		No (*n* = 423)	Yes (*n* = 152)
Baseline FEV1	88.5 (18.4)	87.9 (18.7)	90.0 (17.3)
Admission FEV1	71.4 (19.3)	71.2 (19.4)	71.8 (19.2)
Midway FEV1	80.5 (19.6)	79.7 (19.8)	82.7 (19.1)
Discharge FEV1	83.3 (19.2)	82.6 (19.3)	85.2 (19.0)
Follow-up FEV1	75.1 (21.7)	74.0 (21.8)	78.1 (21.2)

Hospital encounters of PwCF not taking modulators were associated with less significant improvements in mean FEV1pp from admission at both midway and discharge (15.6% vs. 18.3%, 19.9% vs. 22.5%, no modulator vs. modulator groups, respectively) (Table 3). Hospitalizations were associated with lung function recovery by discharge regardless of modulator use. Follow-up visits ranging from 1 week to 13 months after hospital care were associated with a mean loss of lung function from discharge (Table 3). Hospitalizations of PwCF taking modulators had smaller reductions in lung function at follow-up. Moreover, while the prevalence of significant improvement in lung function at discharge was similar regardless of modulator use (68.1% vs. 66.7%, no modulator vs. modulator, respectively), at follow-up there were differences based on modulator use in the prevalence of significant sustained improvement in lung function from discharge (51.1% vs. 59.6%, no modulator vs. modulator groups, respectively) (Supplementary Table S2). Differences in the prevalence of significant improvements in FEV1pp at follow-up by modulator use were larger among hospitalizations of PwCF 12 years and older compared with those of younger PwCF (Supplementary Table S3).

**Table 3:** Mean % change and standard deviation in FEV1pp at key timepoints, overall and by modulator use (yes vs. no).

Time interval	Total (*N* = 575)	Modulator therapy	
		No (*n* = 423)	Yes (*n* = 152)
Admission: % change from baseline	−20.2 (12.3)	−20.0 (11.9)	−20.4 (13.5)
Midway: % change from admission	16.3 (19.2)	15.6 (17.7)	18.3 (22.7)
Discharge: % change from admission	20.6 (21.4)	19.9 (19.0)	22.5 (26.9)
Follow-up: % change from discharge	−10.1 (17.7)	−11.0 (14.6)	−7.5 (24.0)
Follow-up: % change from baseline	−16.0 (15.1)	−16.8 (14.8)	−13.9 (15.6)

Changes in FEV1pp from pre-admission baseline were also examined. Mean FEV1pp was lower at follow-up than pre-admission baseline for all encounters (Table 2). At admission, the mean % relative decrease in FEV1pp from baseline was similar regardless of modulator use (−20.0 vs. −20.4, no modulator vs. modulator groups, respectively) (Table 3). There was a mean loss of lung function from pre-admission baseline at follow-up (−13.9 vs. −16.8, no modulator vs. modulator groups, respectively) (Table 3). At follow-up, there also were differences based on modulator use in the prevalence of significant loss of lung function from baseline (66.5% vs. 53.0%, no modulator vs. modulator groups, respectively) (Supplementary Table S2). This difference was larger among hospitalizations of PwCF 12 years and older (Supplementary Table S3).

Additionally, trends in FEV1pp persisted after conducting regression analyses to adjust for confounding by differences in baseline characteristics. Standardized differences in hospital encounters with and without modulators can be seen in Supplement Table S4. In these analyses, hospitalizations of PwCF taking modulators were not associated with an increased probability of significant improvement at midway or discharge assessments. However, they were associated with a significantly higher probability of sustained improvement in FEV1pp from discharge (difference in probabilities 0.119, *p* < 0.05) and a lower probability of loss of lung function from baseline (difference in probabilities −0.123, *p* < 0.05) at follow-up (Table 4).

**Table 4:** Potential probability of each outcome and average treatment effect in the population (ATE) according to modulator use (yes vs. no)^a^.

Lung function outcome	Probability of outcome (95% CI)		Difference in	*p*-value^b^
	No modulator	Modulator	Probabilities (95% CI)	
MW: significant improvement	0.553 (0.500, 0.606)	0.539 (0.456, 0.622)	−0.014 (−0.104, 0.076)	0.761
DC: significant improvement	0.675 (0.621, 0.729)	0.633 (0.557, 0.710)	−0.042 (−0.126, 0.042)	0.329
FU: sustained improvement	0.512 (0.441, 0.583)	0.631 (0.554, 0.709)	0.119 (0.026, 0.213)	0.012
FU: loss of function	0.670 (0.607, 0.733)	0.548 (0.455, 0.642)	−0.123 (−0.218, −0.026)	0.013

CI, confidence interval; DC, discharge; FU, follow-up; and MW, midway.

^a^ Potential outcome probabilities for each modulator group and their difference are from IPTW regression models. Cluster robust standard errors were used to account for some patients being represented more than once in our study sample.

^b^ *p*-value for difference in potential outcome probabilities between groups.

Sensitivity analyses were performed looking only at encounters for PwCF with dF508 homozygous genetics. In this sub-group, hospitalizations of PwCF taking modulators were associated with a higher probability of sustained improvement in FEV1pp from discharge at follow-up (difference in probabilities 0.108, *p* < 0.068) and a reduced probability of loss of FEV1pp from baseline at follow-up (difference in probabilities −0.076, *p* = 0.181) (Supplementary Table S5). Additionally, because almost a third of hospitalizations used midway lung function assessments as discharge assessments, a sub-analysis used data only from hospitalizations in which midway assessments were at least 3 days prior to discharge assessments. In this analysis, again there was no significant difference in the probability of a significant improvement in lung function at the midway assessment based on modulator use (Supplementary Table S6).

An additional sensitivity analysis was performed to account for the 13 PwCF (9% of the study cohort) who represented 25% of the PEx-related hospitalizations. Of these, seven PwCF accounted for 32% of hospital encounters of PwCF prescribed modulators, and six PwCF accounted for 23% of hospital encounters of PwCF not taking modulators. Hospitalizations of these 13 PwCF with frequent hospitalizations were associated with lower rates of sustained improvement from discharge and higher rates of loss of lung function from baseline at follow-up, potentially influencing the overall study results. Omitting these encounters, hospitalizations of PwCF taking modulators had a higher probability of sustained improvement in lung function (difference in probabilities 0.107, *p* < 0.05) and reduced probability of loss of lung function from baseline (difference in probabilities −0.145, *p* < 0.05) at follow-up (Supplementary Table S7).

## Discussion

This study explored the effect of any CFTR modulator therapy on lung function recovery for PwCF hospitalized for PEx. Hospitalizations for PwCF taking modulators were associated with higher lung function at baseline, midway, discharge, and follow-up, consistent with known benefits of modulators (2, 4–7). Inpatient treatment for PEx was associated with lung function recovery at discharge for hospitalizations regardless of modulator use.

In this study, lung function recovery at discharge was followed by loss of lung function at follow-up that was partially ameliorated by taking any modulator. Data revealed significant differences in the probability of sustained lung function improvement and loss of lung function at follow-up based on modulator use. These differences persisted when analyses were limited to PwCF with dF508 homozygous genetics and when hospitalizations of PwCF with frequent hospitalizations during the study period were not included. Small differences in probabilities and their statistical significance in these analyses can be explained by small sample sizes as well as the range in clinical response to CF modulators (2–6, 8, 9). Despite that 82.9% of the PEx encounters in this study were of PwCF taking two-drug combination modulators associated with lower rates of CFTR restoration, findings revealed that taking any modulator therapy was associated with sustained FEV1pp recovery after PEx. A secondary analysis of Standardized Treatment of Pulmonary Exacerbations 2 (STOP2) trial data revealed no differences in the change in FEV1pp following intravenous antibiotics for PEx in adults taking two-drug combination modulators (20). Long-term effects on FEV1 trajectories following treatment for PEx based on modulator use were not explored in that study, however.

While length of stay was not different based on modulator use in this study, there was a significant improvement in mean FEV1 by midway assessment for all hospitalizations. Future investigations of lung function outcomes based on duration of therapy for PEx for PwCF taking modulators are needed (14). The STOP2 trial revealed that prolonged antibiotic therapy was not superior to shorter courses for adults with CF (20). In our study, during approximately one-third of hospitalizations, PwCF were discharged within 3 days of their midway assessment, suggesting that improvements within the first week were considered adequate. Additionally, larger improvements at the midway assessment were seen in hospitalizations of PwCF taking modulators. Possibly, this finding would be more significant in a population with higher ETI use. This is important given the substantial cost and burden of care associated with inpatient treatment for PEx. Limited data suggest that obtaining midway lung function data earlier in a hospitalization is associated with a shorter length of stay (15, 21, 22). In this study, there was variability in the timing of midway assessments, preventing further analyses on the optimal length of therapy.

Data also demonstrated a mean loss of lung function from pre-admission baseline at follow-up despite modulator use, with approximately half of hospitalizations of PwCF taking modulators having a significant decline from baseline. This contrasts with previous work suggesting that 12%–35% of PwCF fail to recover baseline lung function after PEx (16, 23–25). Variable definitions of baseline FEV1pp and the wide range in timing of follow-up visits in this study likely explain these differences. Since some PwCF only present to the clinic when sick and visits 1 month or more following hospitalization were included as follow-up visits in this study, follow-up FEV1pp values included assessments made during sick visits several months after a hospitalization. Similarly, for some, the best FEV1pp in the year prior to a hospitalization may have represented a discharge FEV1 from a prior hospitalization rather than an outpatient assessment when well. In this study, hospitalizations were associated with relative declines in FEV1pp from baseline at admission that were similar regardless of modulator use (20.2% overall) and larger than previously reported (mean 12% absolute decline in FEV1pp) (16). In addition to variable definitions of baseline FEV1pp, poor adherence with modulator therapy or airway clearance therapy prior to hospitalization likely contributed to these findings.

In addition to FEV1pp findings, data also showed a significant difference in BMI between the two groups of hospitalizations, aligning with an understanding of the effects of modulators on nutritional status for PwCF (2, 4, 6, 26). There was no difference in rates of PA colonization. Rates of PA were high, which is likely reflective of its association with inpatient treatment of PEx (10–12) and not representative of the CF population overall. While treatment with CFTR modulators has been associated with decreases in CF pathogen abundance, most PwCF remain infected with pathogens present prior to modulator treatment (23, 27, 28). Treatment with ETI early in life may change the natural progression of pathogen colonization in CF.

There are several limitations to this study. Comparisons of hospital encounters for PwCF taking two-drug combination vs. three-drug combination modulator therapy were not assessed due to the small sample size. Similarly, stratified analyses based on specific modulators could not be completed due to small sample size. Because of the age of the study population and age-based changes in eligibility criteria for modulator therapy during the study period, 82.9% of the hospital encounters associated with modulator use in this cohort were of PwCF taking two-drug combination products, modulators associated with lower restoration of CFTR (4, 5, 26). Differences in lung function for hospitalizations of PwCF with and without modulators would likely be magnified in an investigation of outcomes for hospitalizations of PwCF taking ETI, particularly if started at a young age (26, 29). Only a small number of PwCF had multiple hospital encounters during the study period including hospitalizations before and after starting modulator use. Given the small numbers, meaningful comparisons of lung function for individual PwCF across different hospital encounters with and without modulators were not possible. Adherence to prescribed modulator therapies was not assessed in this review and may affect baseline and follow-up FEV1pp assessments. Similarly, the length of time on modulator therapy was variable, with some PwCF starting modulators during a hospitalization, affecting admission, midway, and possibly discharge FEV1pp. Definitions of baseline FEV1pp (best in year prior to hospitalization) may have led to the use of falsely elevated baseline FEV1pp. The study population of this single-center study, with high rates of PA and frequent hospitalizations, may not be representative of PwCF overall. The effects of various antimicrobial regimens on lung function recovery during and after PEx were not explored in this study, although the modulator vs. no modulator groups did not differ significantly by sputum culture results (Table 1), suggesting that antibiotic choices were likely to be similar. Variation in availability and timing of lung function assessments contributed to missing data points across hospitalizations, limiting some analyses. While 1-month hospital follow-up is routinely recommended, the timing of follow-up visits was variable. Approximately 20%–25% hospitalizations with and without modulators were associated with steroid use, possibly affecting midway and discharge lung function assessments (30). Moreover, this study did not investigate the effect of modulators on the risk of PEx for PwCF. The COVID-19 pandemic may have affected rates of hospitalization for PEx during the study period (31). Moreover, the use of %predicted values and NHANES race-based normative data rather than *z*-scores and race-neutral Global Lung Initiative normative data reflects the pulmonary function test laboratory standards during the study period and is no longer recommended (32, 33).

This study is the first to describe the effect of modulators on lung function recovery following PEx. Data from this study add to our knowledge of the benefits and limitations of modulator therapy in PwCF. In this study, hospitalizations of PwCF taking any modulator were associated with higher baseline lung function and improved lung function recovery by discharge. In addition, hospitalizations of PwCF taking any modulator were associated with a significantly higher probability of sustained improvement in lung function from discharge and a lower probability of loss of lung function from baseline at follow-up. The Streamlined Treatment of Pulmonary Exacerbations in Pediatrics (NCT04608019) study suggests that the need for oral antimicrobials for mild PEx in children with CF will be reduced with ETI use (34). Further studies are needed to elucidate their effect on inpatient treatment for PEx and lung function trajectories of PwCF taking ETI starting early in life.

## Data Availability

The raw data supporting the conclusions of this article will be made available by the authors, without undue reservation.
