# Supplementary material for: The effect of modulators on lung function following inpatient treatment for CF exacerbations
Source: Front Pediatr. 2025 Aug 21;13:1654122. doi: 10.3389/fped.2025.1654122 (PMC12408314; doi:10.3389/fped.2025.1654122)
Supplement: Supplementary file 1 [file Supplementaryfile1.docx]

**Supplement**

**Table S1**. Availability of CFTR modulators in the United States before and during the study period (2014-2021).^1^

| **Modulator Name** | **Data of US-FDA approval** | **CFTR mutations** | **Ages** |
| --- | --- | --- | --- |
| Ivacaftor | January 2012  2014  March 2015  2017  August 2018  December 2020 | G551D  Additional 8 gating mutations, R117H  Additional 28 residual function mutations  Additional 59 mutations | ≥6 years  ≥2 years  ≥12 months  ≥4 months |
| Lumacaftor/ivacaftor | July 2015  September 2016  August 2018 | Homozygous F508del | ≥12 years  ≥6 years  ≥2 years |
| Tezacaftor/ivacaftor | February 2018  December 2020 | Homozygous F508del or one of 26 responsive mutations  Additional 127 mutations | ≥12 years  ≥6 years |
| Elexacaftor/tezacaftor/ivacaftor | October 2019  December 2020 | At least one copy F508del  Additional 177 mutations | ≥12 years |

^1^Modified from Goetz DM, Savant AP. Review of CFTR Modulators 2020. *Pediatr. Pulmonol.* 2021;1‐12.[29]

**Table S2.** Prevalence of each outcome: Improvement at mid-way, improvement at discharge, sustained improvement, and loss of function, overall and by modulator use (yes vs. no)

|  |  | **Modulator Therapy** | |
| --- | --- | --- | --- |
|  | **Total** | **No** | **Yes** |
| Mid-way: Significant improvement^1^ |  |  |  |
| No | 243 (43.8%) | 180 (44.1%) | 63 (42.9%) |
| Yes | 312 (56.2%) | 228 (55.9%) | 84 (57.1%) |
| Discharge: Significant improvement^2^ |  |  |  |
| No | 179 (32.3%) | 130 (31.9%) | 49 (33.3%) |
| Yes | 376 (67.7%) | 278 (68.1%) | 98 (66.7%) |
| Follow-up: Sustained improvement^3^ |  |  |  |
| No | 265 (46.7%) | 204 (48.9%) | 61 (40.4%) |
| Yes | 303 (53.3%) | 213 (51.1%) | 90 (59.6%) |
| Follow-up: Loss of function^4^ |  |  |  |
| No | 211 (37.1%) | 140 (33.5%) | 71 (47.0%) |
| Yes | 358 (62.9%) | 278 (66.5%) | 80 (53.0%) |

^1^ N=20 missing FEV1 at admission or midway; n=15 no modulator encounters; n=5 modulator encounters

^2^ N=20 missing FEV1 at admission or discharge; n=15 no modulator encounters; n=5 modulator encounters

^3^ N=7 missing FEV1 at discharge or follow-up; n=6 no modulator encounters; n=1 modulator encounter

^4^ N=6 missing FEV1 at baseline or follow-up; n=5 no modulator encounters; n=1 modulator encounter

**Table S3.** Prevalence of each outcome according to age group: Improvement at mid-way, improvement at discharge, sustained improvement, and loss of function, overall and by modulator use (yes vs. no)

|  | **5-12 years** | | **12+ years** | |
| --- | --- | --- | --- | --- |
|  | **No Modulator**  **(n=101)** | **Yes Modulator**  **(n=26)** | **No Modulator**  **(n=322)** | **Yes Modulator**  **(n=126)** |
| Mid-way: Significant improvement^1^ |  |  |  |  |
| No | 60 (60.6%) | 13 (50.0%) | 120 (38.8%) | 50 (41.3%) |
| Yes | 39 (39.4%) | 13 (50.0%) | 189 (61.2%) | 71 (58.7%) |
| Discharge: Significant improvement^2^ |  |  |  |  |
| No | 39 (39.4%) | 10 (38.5%) | 91 (29.4%) | 39 (32.2%) |
| Yes | 60 (60.6%) | 16 (61.5%) | 218 (70.6%) | 82 (67.8%) |
| Follow-up: Sustained improvement^3^ |  |  |  |  |
| No | 32 (32.0%) | 7 (26.9%) | 172 (54.3%) | 54 (43.2%) |
| Yes | 68 (68.0%) | 19 (73.1%) | 145 (45.7%) | 71 (56.8%) |
| Follow-up: Loss of function^4^ |  |  |  |  |
| No | 42 (42.4%) | 12 (46.2%) | 98 (30.75) | 59 (47.2%) |
| Yes | 57 (57.6%) | 14 (53.8%) | 221 (69.3%) | 66 (52.8%) |

^1^ N=20 missing FEV1 at admission or midway; n=15 no modulator encounters; n=5 modulator encounters

^2^ N=20 missing FEV1 at admission or discharge; n=15 no modulator encounters; n=5 modulator encounters

^3^ N=7 missing FEV1 at discharge or follow-up; n=6 no modulator encounters; n=1 modulator encounter

^4^ N=6 missing FEV1 at baseline or follow-up; n=5 no modulator encounters; n=1 modulator encounter

**Table S4**. Comparison of characteristics in hospitalizations with and without a modulator using standardized differences.

|  | **Standardized Difference** | |
| --- | --- | --- |
|  | Raw | IPTW-Weighted |
| ***Patient characteristics*** |  |  |
| Biologic Sex (male vs. female) | -0.341 | 0.034 |
| Age at admission (years) | 0.149 | -0.020 |
| Body Mass Index (kg/m^2^) | 0.269 | -0.024 |
| Race (non-White vs. White) | -0.023 | 0.000 |
| ***Sputum culture characteristics*** |  |  |
| PA (yes vs. no) | 0.068 | 0.046 |
| ***Hospitalization characteristics*** |  |  |
| Steroid treatment (yes vs. no) | -0.139 | -0.025 |
| # PEx admissions in past year | 0.024 | 0.033 |
| Length of stay (days) | -0.010 | -0.006 |
| Home therapy (yes vs. no) | -0.028 | 0.041 |
| ***PEx severity*** |  |  |
| % change FEV1pp from baseline at admission | -0.035 | 0.002 |

**Table S5.** F508/F508 only: Potential probability of each outcome according to modulator use

|  | ***Probability of Outcome (95% CI)^1^*** | | ***Difference in*** |  |
| --- | --- | --- | --- | --- |
|  | ***No Modulator*** | ***Modulator*** | ***Probabilities (95% CI)*** | ***p-value^2^*** |
| MW: Significant improvement | 0.569 (0.497, 0.641) | 0.549 (0.450, 0.647) | -0.020 (-0.131, 0.090) | 0.720 |
| DC: Significant improvement | 0.684 (0.604, 0.764) | 0.651 (0.569, 0.733) | -0.033 (-0.129, 0.063) | 0.500 |
| FU: Sustained improvement | 0.506 (0.409, 0.603) | 0.614 (0.522, 0.706) | 0.108 (-0.008, 0.224) | 0.068 |
| FU: Loss of function | 0.664 (0.579, 0.749) | 0.587 (0.474, 0.701) | -0.076 (-0.189, 0.036) | 0.181 |

Abbreviations: CI, confidence interval; DC, discharge; FU, follow-up; and MW, mid-way

^1^ Potential outcome probability for each modulator group

^2^ p-value for difference in potential outcome probabilities between groups

**Table S6.** True Mid-way: Potential probability of each outcome according to modulator use

|  | ***Probability of Outcome (95% CI)^1^*** | | ***Difference in*** |  |
| --- | --- | --- | --- | --- |
|  | ***No Modulator*** | ***Modulator*** | ***Probabilities (95% CI)*** | ***p-value^2^*** |
| MW: Significant improvement | 0.485 (0.422, 0.549) | 0.499 (0.388, 0.610) | 0.013 (-0.104, 0.130) | 0.826 |

Abbreviations: CI, confidence interval; MW, mid-way

^1^ Potential outcome probability for each modulator group

^2^ p-value for difference in potential outcome probabilities between groups

**Table S7.** Without influential patients: Potential probability of each outcome according to modulator use

|  | ***Probability of Outcome (95% CI)^1^*** | | ***Difference in*** |  |
| --- | --- | --- | --- | --- |
|  | ***No Modulator*** | ***Modulator*** | ***Probabilities (95% CI)*** | ***p-value^2^*** |
| MW: Significant improvement | 0.520 (0.455, 0.584) | 0.516 (0.419, 0.614) | -0.004 (-0.106, 0.099) | 0.946 |
| DC: Significant improvement | 0.639 (0.579, 0.699) | 0.606 (0.522, 0.689) | -0.034 (-0.133, 0.066) | 0.509 |
| FU: Sustained improvement | 0.581 (0.509, 0.654) | 0.689 (0.601, 0.777) | 0.107 (0.004, 0.211) | 0.041 |
| FU: Loss of function | 0.598 (0.533, 0.664) | 0.454 (0.353, 0.554) | -0.145 (-0.258, -0.032) | 0.012 |

Abbreviations: CI, confidence interval; DC, discharge; FU, follow-up; and MW, mid-way

^1^ Potential outcome probability for each modulator group

^2^ p-value for difference in potential outcome probabilities between groups
